# Inferior Prefrontal Cortex Mediates the Relationship between Phosphatidylcholine and Executive Functions in Healthy, Older Adults

**DOI:** 10.3389/fnagi.2016.00226

**Published:** 2016-09-28

**Authors:** Marta K. Zamroziewicz, Chris E. Zwilling, Aron K. Barbey

**Affiliations:** ^1^Decision Neuroscience Laboratory, University of Illinois Urbana-Champaign, UrbanaIL, USA; ^2^Beckman Institute for Advanced Science and Technology, University of Illinois Urbana-Champaign, UrbanaIL, USA; ^3^Neuroscience Program, University of Illinois Urbana-Champaign, UrbanaIL, USA; ^4^Department of Psychology, University of Illinois Urbana-Champaign, UrbanaIL, USA; ^5^Department of Speech and Hearing Science, University of Illinois Urbana-Champaign, UrbanaIL, USA; ^6^Department of Internal Medicine, University of Illinois Urbana-Champaign, UrbanaIL, USA; ^7^Carl R. Woese Institute for Genomic Biology, University of Illinois Urbana-Champaign, UrbanaIL, USA; ^8^Carle Neuroscience Institute, Carle Foundation Hospital, UrbanaIL, USA

**Keywords:** phosphatidylcholine, inferior prefrontal cortex, executive functions, cognitive aging, nutritional cognitive neuroscience

## Abstract

**Objectives:** This study examines the neural mechanisms that mediate the relationship between phosphatidylcholine and executive functions in cognitively intact older adults. We hypothesized that higher plasma levels of phosphatidylcholine are associated with better performance on a particular component of the executive functions, namely cognitive flexibility, and that this relationship is mediated by gray matter structure of regions within the prefrontal cortex (PFC) that have been implicated in cognitive flexibility.

**Methods:** We examined 72 cognitively intact adults between the ages of 65 and 75 in an observational, cross-sectional study to investigate the relationship between blood biomarkers of phosphatidylcholine, tests of cognitive flexibility (measured by the Delis–Kaplan Executive Function System Trail Making Test), and gray matter structure of regions within the PFC. A three-step mediation analysis was implemented using multivariate linear regressions and we controlled for age, sex, education, income, depression status, and body mass index.

**Results:** The mediation analysis revealed that gray matter thickness of one region within the PFC, the left inferior PFC (Brodmann’s Area 45), mediates the relationship between phosphatidylcholine blood biomarkers and cognitive flexibility.

**Conclusion:** These results suggest that particular nutrients may slow or prevent age-related cognitive decline by influencing specific structures within the brain. This report demonstrates a novel structural mediation between plasma phosphatidylcholine levels and cognitive flexibility. Future work should examine the potential mechanisms underlying this mediation, including phosphatidylcholine-dependent cell membrane integrity of the inferior PFC and phosphatidylcholine-dependent cholinergic projections to the inferior PFC.

## Introduction

A rapidly expanding older adult population has produced significant medical and economic demands for the treatment and care of individuals with age-related health disorders that continue to rise. The prevalence of Alzheimer’s disease, for example, is projected to increase in the United States from 5.1 to 13.2 million by 2050, and associated healthcare expenditures are estimated to surpass one trillion dollars (Alzheimer’s Association, 2013). Therefore, establishing a successful strategy to promote healthy brain aging is of great interest to public health efforts and the United States economy. Nutrition and the many bioactive substances present in the diet have been increasingly recognized as a promising target for intervention efforts to promote healthy brain aging (Zamroziewicz and Barbey, 2016). Identifying the means through which dietary intake may influence brain health will guide the development of successful dietary strategies for healthy brain aging.

Accumulating evidence suggests that phosphatidylcholine is a robust marker of age-related membrane degeneration and is associated with cognitive decline (Zeisel, 2006; Frisardi et al., 2011; Mapstone et al., 2014; Whiley et al., 2014; Norris et al., 2015; Wurtman, 2015). Phosphatidylcholine is a phospholipid that carries a choline head group (Li and Vance, 2008). Phosphatidylcholine found in the blood may be derived from dietary sources, or may be endogenously synthesized by the phosphatidylethanolamine *N*-methyltransferase (PEMT) pathway (Zeisel, 2006). Phosphatidylcholine serves a neuroprotective role by providing an essential component of neuronal membranes and a significant portion of the total choline pool, which contributes to forebrain cholinergic projections (Frisardi et al., 2011). However, the core brain regions upon which phosphatidylcholine may act are unknown. This study aims to investigate the neural structures that mediate the relationship between plasma phosphatidylcholine levels and an important aspect of cognitive aging, decline in a component of the executive functions known as cognitive flexibility.

Low plasma phosphatidylcholine levels are highly predictive of cognitive decline, and low levels of important components of phosphatidylcholine, including the long-chain polyunsaturated fatty acid docosahexaenoic acid (DHA) and choline, are predictive of age-related decline in executive functions (Beydoun et al., 2007; Bowman et al., 2012; Nurk et al., 2013; Witte et al., 2013; Naber et al., 2015). Executive functions traditionally consist of planning and execution of goal-directed behaviors, abstract reasoning, and judgment, but also reflect the efficiency with which an individual applies his or her knowledge to cope with everyday life (Stuss and Alexander, 2000; Princiotta and Devries, 2014). Within the continuum of normal aging or preclinical stages of dementia, the presence of executive dysfunction may occur without measurable deficits in general cognition. Therefore, executive dysfunction may be a robust early marker of cognitive decline (Johnson et al., 2007). Importantly, components of phosphatidylcholine, including long-chain polyunsaturated fatty acids and choline, have been associated with prefrontal cortical integrity and forebrain cholinergic projections, respectively, suggesting a link between phosphatidylcholine and the PFC-driven executive functions (Kolisnyk et al., 2013; Zamroziewicz et al., 2015). More specifically, long-chain polyunsaturated fatty acids have been shown to influence cognitive flexibility, a component of the executive functions (Bowman et al., 2013; Johnston et al., 2013; Zamroziewicz et al., 2015). Cognitive flexibility refers to the ability to adjust to new demands or rules, and can be measured using task switching paradigms (Diamond, 2013).

Executive functions are implemented within the prefrontal cortex (PFC), and particular aspects of the executive functions may be localized to specific sub-regions within the PFC (Barbey et al., 2012, 2013a,b,c, 2014a,b). Larger gray matter thickness and volume in the PFC has been associated with better performance on tasks that elicit executive functions (Kochunov et al., 2009; Burzynska et al., 2012; Tu et al., 2012; Yuan and Raz, 2014). For example, the inferior PFC has been implicated in the cognitive control of memory, including semantic retrieval, recollection of contextual details about past events, resolution of proactive interference in working memory, and task switching (Badre and Wagner, 2007). The inferior PFC is particularly susceptible to age-related cortical thinning, and age-related changes in cholinergic projections (Aron et al., 2004; Fjell et al., 2009). Integrity of the left inferior PFC has been linked to cognitive flexibility, as measured by task switching paradigms (Aron et al., 2004).

In summary, prior research indicates that: (i) phosphatidylcholine is highly predictive of age-related cognitive decline; (ii) cognitive flexibility is an early marker of cognitive decline amenable to the effects of phosphatidylcholine components; and (iii) particular regions within the PFC, such as the inferior PFC, are critical for cognitive flexibility and susceptible to age-related degeneration. Therefore, we examined the role of regions within the PFC in mediating the relationship between plasma phosphatidylcholine and cognitive flexibility in cognitively intact aging individuals.

## Materials and Methods

### Participants

This cross-sectional study enrolled 122 elderly adults from Carle Foundation Hospital, a local and readily available cohort of well-characterized elderly adults. No participants were cognitively impaired, as defined by a score of lower than 26 on the Mini-Mental State Examination (Folstein et al., 1975). Participants with a diagnosis of mild cognitive impairment, dementia, psychiatric illness within the last 3 years, stroke within the past 12 months, and cancer within the last 3 years were excluded. Participants were also excluded for current chemotherapy or radiation, an inability to complete study activities, prior involvement in cognitive training or dietary intervention studies, and contraindications for magnetic resonance imaging (MRI). Of these 122 participants, 72 subjects had a complete dataset at time of data analysis, including neuropsychological testing, MRI, and blood biomarker analysis.

### Standard Protocol Approval and Participant Consent

This study was approved by the University of Illinois Institutional Review Board and the Carle Hospital Institutional Review Board and, in accordance with the stated guidelines, all participants read and signed informed consent documents.

### Biomarker Acquisition and Analysis

Plasma was spiked with stable labeled internal standards of all the analytes, and extracted using the method modified from Bligh and Dyer (1959). Samples were extracted with methanol/chloroform (2:1, v/v). The mixture was vortexed and left at -20°C overnight. At the end of the extraction with methanol/chloroform, samples were centrifuged and supernatants transferred into new microcentrifuge tubes. Residues were re-extracted with methanol/chloroform/water (2:1:0.8, v/v/v). After vigorous vortexing and centrifugation, supernatants were collected and combined with the first extract. Water and chloroform were added into the resulting solutions to allow for phase separation. After centrifugation, the organic phase, which contains phosphatidylcholine, was 1:10 diluted with methanol and transferred into HPLC vials for instrumental analysis.

Quantification of the analytes was performed using liquid chromatography-stable isotope dilution-multiple reaction monitoring mass spectrometry (LC-SID-MRM/MS). Chromato-graphic separations were performed on an Atlantis Silica HILIC 3 μm 4.6 × 50mm column (Waters Corp, Milford, CT, USA) using a Waters ACQUITY UPLC system. The column was heated to 40°C, and the flow rate maintained at 1 mL/min. The mobile phases were: A – 10% acetonitrile/90% water with 10 mM ammonium formate and 0.125% formic acid, and B – 90% acetonitrile/10% water with 10 mM ammonium formate and 0.125% formic acid. For organic analytes, the gradient was at 5% A for 0.05 min, to 20% A in 2.95 min, to 55% A in 0.05 min, at 55% A in 0.95 min, to 5% A in 0.05 min, and at 5% A for 2.95 min. The analytes and their corresponding isotopes were monitored on a Waters TQ detector using characteristic precursor-product ion transitions. Concentrations of each analyte in the samples were determined using the peak area ratio of the analyte to its isotope. MS parameters for phosphatidylcholine were as follows: precursor at 193 m/z, product at 193 m/z. Phosphatidylcholine levels were included in analyses as a continuous variable.

### Neuropsychological Tests

Executive functions were measured by the Delis–Kaplan Executive Function System (D–KEFS) Trail Making Test (Delis et al., 2006). This assessment yields a measure of the executive functions that can be isolated from underlying skills, including visual scanning, number sequencing, letter sequencing, and motor speed. In this task, participants alternate between multiple task goals (either number or letter sequencing), which elicits a specific component of the executive functions known as cognitive flexibility. The reported results from the D-KEFS Trail Making Test assess cognitive flexibility while controlling for number and letter sequencing trials and therefore provide a measure of cognitive flexibility that is not confounded by underlying cognitive abilities (i.e., number and letter sequencing) required by the task.

### Volumetric Brain MRI

Volumetric analysis was performed on data from a 3D high-resolution (0.9 mm isotropic) T1-weighted scan using MPRAGE acquisition. Cortical reconstruction was performed with the Freesurfer image analysis suite, which is documented and freely available for download online^1^ The technical details of these procedures are described in prior publications (Dale and Sereno, 1993; Dale et al., 1999; Fischl et al., 1999a,b, 2001, 2002, 2004; Fischl and Dale, 2000; Fischl, 2004; Ségonne et al., 2004; Han et al., 2006; Jovicich et al., 2006; Reuter et al., 2010, 2012). All cortical reconstructions were manually checked for accuracy, as recommended by the software developers. This analysis focused on gray matter thickness in the PFC provided by Freesurfer parcellation. These regions included the superior frontal cortex, rostral middle frontal cortex, the caudal middle frontal cortex, pars opercularis, pars triangularis, pars orbitalis, lateral orbitofrontal cortex, medial orbitofrontal cortex, precentral gyrus, paracentral gyrus, frontal pole, rostral anterior cingulate cortex, and caudal anterior cingulate cortex.

### Covariates

Covariates previously associated with cognitive decline (Coffey et al., 1998, 1999; Fotenos et al., 2008; Gunstad et al., 2008; Raz et al., 2010; van Tol et al., 2010) were tested, including age (continuous), gender (nominal, man/woman), education (ordinal, five fixed levels), income (ordinal, six fixed levels), body mass index (continuous, BMI), and depression status (nominal, yes/no). Although all participants had received a diagnosis of no depression at enrollment, the SF-36 Health Survey (Ware et al., 1993) revealed five participants with symptoms consistent with depression and so, in accordance with other studies, this was considered in the analysis as a covariate. PFC gray matter thickness (continuous) was also included as a covariate in mediation analyses to assess the relationship between specific regions within the PFC, plasma phosphatidylcholine, and cognitive flexibility. Covariates were included in each of the three steps of the mediation analysis.

### Statistical Analyses

A formal mediation analysis was used in an effort to better understand the relationship between phosphatidylcholine levels, gray matter thickness of regions within the PFC, and cognitive flexibility using a three-step framework. The goal of the mediation analysis was to understand whether the relationship between phosphatidylcholine levels and cognitive flexibility was mediated by gray matter thickness of regions within the PFC. The primary requirement for mediation is a significant indirect mediation effect, or the effect of the independent variable (phosphatidylcholine) through the mediator (gray matter thickness of a PFC region) on the dependent variable (cognitive flexibility) (Zhao et al., 2010).

Statistics were performed in SPSS Statistical Packages version 23 (SPSS, Inc., Chicago, IL, USA), and mediation analyses were performed using the *indirect* macro designed for SPSS (Preacher and Hayes, 2008). Statistics were performed as follows:

(1) In the first step, a regression model was used to characterize the relationship between phosphatidylcholine levels and gray matter thickness of regions in the PFC, controlling for the covariates in Section “Covariates” (path a).
(2) In the second step, a regression model was used to characterize the relationship between phosphatidylcholine levels and cognitive flexibility, controlling for the covariates in Section “Covariates” (path c).
(3) In the third step, the *indirect* macro was used to implement the bootstrapping method to estimate mediation effects. This analysis drew 1000 bootstrapped samples with replacement from the dataset to estimate a sampling distribution for the indirect and direct mediation effects, controlling for the covariates in Section “Covariates.” The indirect mediation effect refers to the pathway from phosphatidylcholine to gray matter thickness of a PFC region to cognitive flexibility (path a–b). The direct mediation effect refers to the direct pathway from phosphatidylcholine to cognitive flexibility (path c’).

A statistically significant mediation that matched the hypothesized framework was indicated by: (i) an indirect mediation effect that did not include zero within 95% bias-corrected confidence intervals, and (ii) a direct mediation effect that did include zero within 95% bias-corrected confidence intervals (Zhao et al., 2010). Results are reported using unstandardized regression coefficients (β) and statistical significance (*p*) for each individual regression relationship, and a 95% bias-corrected confidence interval (95% CI) for the direct and indirect effects of the mediation.

## Results

### Participant Characteristics

Participants had a mean age of 69 years and 64 percent of participants were females. Education levels were reported as follows: 1 percent of participants completed some high school, 14 percent of participants received a high school degree, 18 percent of participants completed some college, and 68 percent of participants received a college degree. Annual household income levels were reported as follows: 1 percent of participants earned less than $15,000, 3 percent of participants earned $15,000 to $25,000, 17 percent of participants earned $25,000 to $50,000, 24 percent of participants earned $50,000 to $75,000, 22 percent of participants earned $75,000 to $100,000, and 33 percent of participants earned over $100,000. The mean phosphatidylcholine level was 2101 μM. The mean D-KEFS Trail Making Test cognitive flexibility score was 8. The mean gray matter thickness of the left PFC was 2.39 mm, and mean gray matter thickness of the left inferior PFC was 2.38 mm (**Table 1**).

**Table 1:** Characteristics of sample^1^.

Demographics	Total *n* = 72
Age (mean years + standard deviation)	69 ± 3
Female, *n*(%)	46(64)
Education, *n*(%)	1(1) some high school
	10(14) high school degree
	12(17) some college
	49(68) college degree
Income, *n*(%)	1(1) < $15,000
	2(3) $15,000 – $25,000
	12(17) $25,000 – $50,000
	17(24) $50,000 – $75,000
	16(22) $75,000 – $100,000
	24(33) > $100,000
Depression, *n*(%)	67(93) no
	5(7) yes
**Plasma nutrients**	**(uM ± std)**
Phosphatidylcholine	2101 ± 400
**Psychometrics**	**(mean ± std)**
Cognitive flexibility score	8 ± 2
**Volumetric MRI (gray matter thickness)**	**(mm± std))**
Left PFC	2.39 ± 0.08
Left inferior PFC	2.38 ± 0.13


*^1^Demographics reflect covariates included in analyses. Plasma nutrients, psychometrics, and volumetric MRI reflect variables of interest included in analyses.*

### Mediation Results

The mediation analyses indicated that out of all regions within the PFC, gray matter thickness of only the left inferior PFC (pars triangularis, Brodmann area 45) mediated the relationship between phosphatidylcholine and cognitive flexibility, corresponding with prior work that suggests an influential role of this region. Each relationship within the mediation is described below in a stepwise fashion.

**FIGURE 1:**
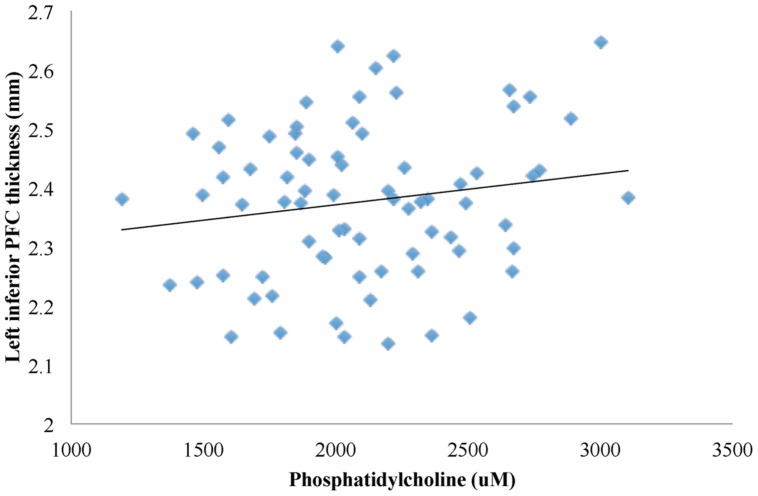
**A linear regression model was used to characterize the relationship between phosphatidylcholine levels and gray matter thickness of regions in the PFC.** Phosphatidylcholine levels positively associated with gray matter thickness of the left inferior PFC (β = 0.001, *p* = 0.007).

**FIGURE 2:**
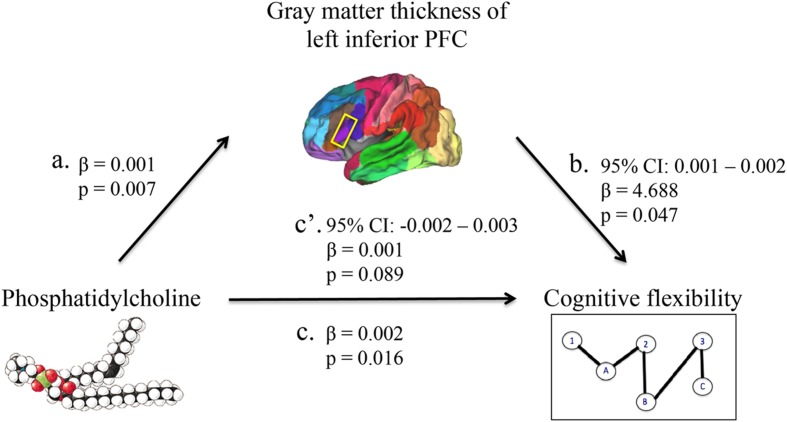
**A mediation model was used to characterize the relationship between phosphatidylcholine levels, gray matter thickness of regions in the PFC, and cognitive flexibility.** Phosphatidylcholine levels positively associated with gray matter thickness of the left inferior PFC (path a). Phosphatidylcholine levels positively associated with cognitive flexibility (path c). The indirect pathway of mediation (i.e., the effect of phosphatidylcholine levels through gray matter thickness of the left inferior PFC on cognitive flexibility; path a–b) was statistically significant. The direct pathway of mediation (i.e., the effect of phosphatidylcholine levels directly on cognitive flexibility, accounting for the effect of gray matter thickness of the left inferior PFC; path c’) was not statistically significant. Therefore, gray matter thickness of the left inferior PFC fully mediated the relationship between phosphatidylcholine levels and cognitive flexibility.

First, higher phosphatidylcholine associated with greater thickness of the left inferior PFC (β = 0.001, *p* = 0.007; **Figures 1** and **2**, path a). Second, higher phosphatidylcholine associated with better cognitive flexibility (β = 0.002, *p* = 0.016, **Figure 2**, path c). Third, the indirect pathway of meditation was significant (95% CI: 0.001 – 0.002, β = 4.688, *p* = 0.047, **Figure 2**, path a–b), but the direct pathway of mediation was insignificant (95% CI: -0.002 – 0.003, β = 0.001, *p* = 0.089, **Figure 2**, path c’). Therefore, the mediation indicated that gray matter thickness of the left inferior PFC fully mediated the relationship between phosphatidylcholine and cognitive flexibility (**Figure 2**).

## Discussion

This study revealed that gray matter thickness of the left inferior PFC mediates the relationship between plasma phosphatidylcholine and cognitive flexibility. The mediation analysis provided a novel finding that links phosphatidylcholine to gray matter integrity of a specific cortical region and a particular component of the executive functions. The individual relationships reported within the mediation, including those between phosphatidylcholine levels and left inferior PFC (**Figure 2**, path a), between phosphatidylcholine levels and cognitive flexibility (**Figure 2**, path c), and between left inferior PFC and cognitive flexibility (**Figure 2**, path b), are each substantiated by prior findings reviewed in turn below.

The first relationship demonstrated a positive association between higher phosphatidylcholine levels and greater thickness in the inferior PFC of the left hemisphere (**Figure 2**, path a). Past studies suggest that phosphatidylcholine plays a critical role in age-related changes in cortical integrity, and the inferior PFC, being a region that thins early in aging, may be particularly susceptible to these effects (Söderberg et al., 1990; Wurtman, 2015). More specifically, phosphatidylcholine may contribute to structure and function of the inferior PFC via cholinergic projections, which enhance functional activity within this region (Blusztajn et al., 1987; Berry et al., 2015). Second, higher phosphatidylcholine levels are associated with better cognitive flexibility (**Figure 2**, path c). Prior work demonstrates that higher phosphatidylcholine levels are related to slower cognitive decline, and components of phosphatidylcholine, including long-chain polyunsaturated fatty acids, such as DHA, and choline, are linked to superior performance on executive function tasks (Schaefer et al., 2006; Beydoun et al., 2007; Bowman et al., 2012; Nurk et al., 2013; Witte et al., 2013; Hartmann et al., 2014; Mapstone et al., 2014; Naber et al., 2015; Zamroziewicz et al., 2015). The indirect pathway of mediation indicated a mediatory effect of left inferior PFC gray matter thickness on the relationship between phosphatidylcholine levels and cognitive flexibility (**Figure 2**, path a–b). Previous studies indicate that greater gray matter thickness within the inferior PFC contributes to superior cognitive flexibility, and that cholinergic transmissions, originating, for example, from phosphatidylcholine-derived choline, underlie activity within the inferior PFC during tasks of cognitive control (Blusztajn et al., 1987; Burzynska et al., 2012; Berry et al., 2015). The unilateral nature of this mediation is supported by prior work, which suggests that regions within the left hemisphere may be selectively susceptible to degeneration and cognitive impairment (Chételat et al., 2005; Querbes et al., 2009; Risacher et al., 2010; Mosconi et al., 2014).

Prior work indicates that the underlying physiological mechanisms of the relationship between phosphatidylcholine levels, cognitive flexibility, and cortical integrity of the inferior PFC may be threefold. First, phosphatidylcholine may help slow or prevent age-related changes in cortical thickness by delivering two molecules that are critical for cortical integrity, including choline and long-chain polyunsaturated fatty acids (Söderberg et al., 1990; Cohen et al., 1995; Zeisel, 2006; Jerneren et al., 2015). Second, the delivery of long-chain polyunsaturated fatty acids may help prevent inflammation in the brain (Wall et al., 2010). Third, delivery of choline contributes to acetylcholine synthesis, a neurotransmitter that has been implicated in set-shifting performance and projects to the inferior PFC via forebrain cholinergic transmissions (Blusztajn et al., 1987; Hasselmo and Sarter, 2011; Berry et al., 2015). Importantly, phosphatidylcholine-derived choline may be a primary contributor to the brain choline pool when age-related changes in brain choline uptake reduce extracellular choline supplies (Zeisel, 2006). Future mechanistic studies are needed to confirm underlying physiological mechanisms of the relationship between phosphatidylcholine levels, cognitive flexibility, and cortical integrity of the inferior PFC.

Research at the frontiers of nutritional cognitive neuroscience seeks to integrate methods that sensitively capture variability in nutritional intake, brain aging, and cognition, and in doing so, elucidate the neural structures that mediate the relationship between nutritional status and cognitive decline. This finding contributes to a growing line of evidence which suggests that particular nutrients may slow or prevent aspects of age-related cognitive decline by influencing specific features of brain aging (Bowman et al., 2012; Zamroziewicz et al., 2015; Gu et al., 2016). In the case of phosphatidylcholine, future studies are needed to assess the origins of plasma phosphatidylcholine, and whether dietary intake or endogenous synthesis preferentially contributes to the neuroprotective effect. Another promising direction for future work is to examine the interactive effects among nutrients through the use of nutrient biomarker pattern analysis – a technique that enables an investigation of the beneficial effects of broader nutrient profiles on healthy brain aging. Ultimately, this line of research can inform clinical investigations of comprehensive and personalized approaches to nutritional intervention that takes into account dietary patterns and individual variability in nutritional status and brain health.

The strengths of the present study include: (i) the use of blood biomarkers to measure physiological status of phosphatidylcholine, (ii) the use of structural magnetic resonance imaging to measure regional cortical integrity with high spatial resolution, and (iii) the assessment of a particular component of cognitive function known to be sensitive to age-related cognitive decline, rather than a global cognitive function measure with little variability in healthy aging adults. Directions for future research include (i) replication of results in a larger sample size, (ii) implementation of a longitudinal study to examine how changes in phosphatidylcholine levels relate to changes in executive functions and integrity of the PFC, (iii) investigation of the physiological mechanisms proposed to underlie the relationship between phosphatidylcholine and PFC structure, (iv) examination of relationship between phosphatidylcholine, executive functions, and PFC integrity in other models, including animal models and clinical populations, (v) elucidation of the relationship between phosphatidylcholine in plasma and cerebrospinal fluid, and (vi) examination of the origins of phosphatidylcholine in blood, as plasma phosphatidylcholine may be derived from the diet or *de novo* synthesis by the phosphatidylethanolamine *N*-methyltransferase (PEMT) pathway (Zeisel, 2006).

## Author Contributions

MZ is the primary author of this manuscript. CZ contributed to drafting and editing of the manuscript. AB is the primary investigator and contributed to drafting and editing of the manuscript.

## Conflict of Interest Statement

The authors declare that the research was conducted in the absence of any commercial or financial relationships that could be construed as a potential conflict of interest.

## Footnotes

1 http://surfer.nmr.mgh.harvard.edu/.
